# Interobserver reliability of the ‘Welfare Quality^®^ Animal Welfare Assessment Protocol for Growing Pigs’

**DOI:** 10.1186/s40064-016-2785-1

**Published:** 2016-07-19

**Authors:** I. Czycholl, C. Kniese, K. Büttner, E. grosse Beilage, L. Schrader, J. Krieter

**Affiliations:** Institute of Animal Breeding and Husbandry, Christian-Albrechts-University, Olshausenstr. 40, 24098 Kiel, Germany; Field Station for Epidemiology, University of Veterinary Medicine Hannover, Foundation, Buescheler Str. 9, 49456 Bakum, Germany; Institute of Animal Welfare and Animal Husbandry, Friedrich Loeffler Institut, Doernbergstr. 25/27, 29223 Celle, Germany

**Keywords:** Interobserver reliability, Welfare Quality^®^, Animal welfare assessment, Pig, Animal-based

## Abstract

The present paper focuses on evaluating the interobserver reliability of the ‘Welfare Quality^®^ Animal Welfare Assessment Protocol for Growing Pigs’. The protocol for growing pigs mainly consists of a Qualitative Behaviour Assessment (QBA), direct behaviour observations (BO) carried out by instantaneous scan sampling and checks for different individual parameters (IP), e.g. presence of tail biting, wounds and bursitis. Three trained observers collected the data by performing 29 combined assessments, which were done at the same time and on the same animals; but they were carried out completely independent of each other. The findings were compared by the calculation of Spearman Rank Correlation Coefficients (RS), Intraclass Correlation Coefficients (ICC), Smallest Detectable Changes (SDC) and Limits of Agreements (LoA). There was no agreement found concerning the adjectives belonging to the QBA (e.g. active: RS: 0.50, ICC: 0.30, SDC: 0.38, LoA: −0.05 to 0.45; fearful: RS: 0.06, ICC: 0.0, SDC: 0.26, LoA: −0.20 to 0.30). In contrast, the BO showed good agreement (e.g. social behaviour: RS: 0.45, ICC: 0.50, SDC: 0.09, LoA: −0.09 to 0.03 use of enrichment material: RS: 0.75, ICC: 0.68, SDC: 0.06, LoA: −0.03 to 0.03). Overall, observers agreed well in the IP, e.g. tail biting (RS: 0.52, ICC: 0.88; SDC: 0.05, LoA: −0.01 to 0.02) and wounds (RS: 0.43, ICC: 0.59, SDC: 0.10, LoA: −0.09 to 0.10). The parameter bursitis showed great differences (RS: 0.10, ICC: 0.0, SDC: 0.35, LoA: −0.37 to 0.40), which can be explained by difficulties in the assessment when the animals moved around quickly or their legs were soiled. In conclusion, the interobserver reliability was good in the BO and most IP, but not for the parameter bursitis and the QBA.

## Background

Animal welfare has become an important subject of political and public discussion (Hobbs et al. 2002). However, the definition of what that term should comprise is very subjective and the discussion is often characterised by a certain amount of emotionality (Broom 1988). That is the reason why there is not only a need for a clear definition, but also for an objective assessment of animal welfare (Webster 2005).

The invention of such a system was the aim of the Welfare Quality^®^ project, during which the ‘Welfare Quality^®^ Animal Welfare Assessment’ protocols were developed. Animal welfare was defined as a multidimensional concept consisting of the absence of thirst, hunger, discomfort, disease, pain and injuries, stress and the expression of normal behaviour (Temple et al. 2011a). This definition was based on the five freedoms of the Farm Animal Welfare Council (FAWC 1993). In the protocols, the implementation of this definition took place in the form of four main principles—good feeding, good housing, good health and appropriate behaviour. In terms of a top–down process, these principles were divided into twelve criteria, which can again be measured by a set of approximately 30 predominantly animal based parameters to be estimated in the stable. After assessment of the parameters in the stable, the measures are usually expressed as percentages of affected animals. From these percentages a dimensionless number between 0 and 100 can be calculated by different mathematical methods, e.g. decision trees as well as I-Spline functions and Choquet Integrals (Welfare Quality^®^^2009^), first at the criteria and afterwards at principle level. Depending on the numbers reached (the closer to 100 the better) the farms are scored and labelled as excellent, enhanced, acceptable or not classified (Welfare Quality^®^^2009^).

The protocols promise to be feasible, valid and reliable, which are basic requirements of an objective measurement method (Velarde and Geers 2007). Feasibility, i.e. a good cost-benefit ratio and the capability of accomplishment is always important for a method to be accepted and implemented into practical conditions. Validity and reliability describe the dependence on a method. In this context, validity outlines the extent to which a parameter assesses what it is supposed to measure and the relevance of that parameter. Reliability implies that the results are repeatable (Velarde and Geers 2007). It is usually divided into the interobserver reliability and the test–retest reliability (de Passille and Rushen 2005). Interobserver reliability means that different trained observers should come to the same conclusions when assessing the same objects at the same time and under the same conditions. Test–retest reliability describes the stability of the measurement method over time, thus in how far results can be reproduced despite minor changes (Martin and Bateson 2007; Windschnurer et al. 2008).

 The assessment of reliability can be carried out with different statistical parameters. In our study, we used the Spearman Rank Correlation Coefficient (RS), the Intraclass Correlation Coefficient (ICC), the Smallest Detectable Change (SDC) as well as the Limits of Agreement (LoA). All these parameters were recommended by de Vet et al. (2006) and were—with exception of the SDC—also used in the animal welfare study on the Test–Retest assessment of the Welfare Quality^®^ protocol by Temple et al. (2013). Each parameter has its own weaknesses and benefits and there is not one single parameter capable of satisfactorily assessing reliability (Dohoo et al. 2003). For this reason, it is often advised to calculate a range of different parameters, namely agreement and reliability parameters, and interpret the reliability of the measured objects based on all statistical coefficients (Dohoo et al. 2003; de Vet et al. 2006; Temple et al. 2012).

In pilot studies, most of the parameters and partially also the criteria included in the Welfare Quality^®^ protocols were tested for their feasibility, validity and reliability (Forkman and Keeling 2009). However, due to the fact that these protocols are relatively new and under the consistent process of improvement and revision, studies on the feasibility, validity and reliability of the entire protocols are rare. Moreover, the few available studies are based mainly on video sequences since on-farm assessment is much more time consuming and costly.

Therefore, the present study analysed the interobserver reliability of the ‘Welfare Quality^®^ Animal Welfare Assessment Protocol for Growing Pigs’ and is thus a first step towards the evaluation of the reliability of the entire protocol. It is one of the first studies to evaluate reliability of the complete protocol carried out by different trained observers on-farm.

## Methods

### Data collection

Data collection was performed between January and August 2013 on 24 German growing pig farms in Lower Saxony and Schleswig–Holstein. The pigs on the farms were housed either conventionally or according to the guidelines of the animal welfare label ‘Tierschutzlabel’ of the German animal welfare organisation ‘Deutscher Tierschutzbund e.V.’ (Tierschutzbund 2013).

On these farms, 29 protocol assessments were carried out by three observers: Observer A and B fulfilled 19 combined assessments, while observer A and C examined ten farms together. During these assessments, the same animals were observed at the same time, but completely independently of each other. The observers had been trained officially by members of the Welfare Quality^®^ project group. Observer agreement was further tested by the evaluation of video sequences and pictures during the study. This was carried out after the first half of data recording in the interobserver reliability study, thus, after the first ten visits of observer A and B as well as the first five visits of observers A and C. It was carried out a second time after completion of data recording of observers A and B and observers A and C, respectively. At all times, more than 85 % of the pictures and videos were sorted into the same categories and therefore good agreement was attained. These were simply control sessions and did not change the rating of observers.

### Ethical statement

The authors declare that the experiments were carried out strictly following international animal welfare guidelines. The institution the authors are affiliated with does not have research ethic committees or review boards (in consultation with the animal welfare officer of the Christian-Albrechts-University, Kiel, Germany). Therefore, the ‘German Animal Welfare Act’ (German designation: TierSchG), the ‘German Order for the Protection of Animals used for Experimental Purposes and other Scientific Purposes (German designation: TierSchVersV) and the ‘German Order for the Protection of Production Animals used for Farming Purposes and other Animals kept for the Production of Animal Products (German designation: TierSchNutztV) were applied. No pain, suffering or injury was inflicted on the animals during the study.

### Protocol assessments

The entire Welfare Quality^®^ protocol, was carried out during each farm visit. The ‘Welfare Quality^®^ Animal Welfare Assessment Protocol for Growing Pigs’ consists of four different parts: a Qualitative Behaviour Assessment (QBA), behaviour observations (BO), a Human Animal Relationship Test (HAR) and the assessment of different individual parameters (IP), which are described in detail below.

#### Qualitative Behaviour Assessment (QBA)

The QBA is the animal based measure that was included in the protocol for the evaluation of positive emotions. Carrying out this behavioural observation method, the observer watches the animals that can be seen well from each observation point for a given time. Thereby, the expressive quality of the animals’ activities is observed at group level. It was carried out on four to six observation points in the stable for a total surveillance time of 20 min. To each of 20 given adjectives, which are (1) active, (2) relaxed, (3) fearful, (4) agitated, (5) calm, (6) content, (7) tense, (8) enjoying, (9) frustrated, (10) bored, (11) playful, (12) positively occupied, (13) listless, (14) lively, (15) indifferent, (16) irritable, (17) aimless, (18) happy, (19) distressed and (20) sociable, a visual analogue scale of 125 mm is assigned. A mark was set on the scale to record whether the observer finds that term to be rather absent (0 mm) or dominant (125 mm) for the animals under study.

For each of the adjectives the length [mm] on the visual analogue scale was measured with a ruler. Thus, for each farm visit, one score in millimetres for each adjective was obtained by each observer. As the whole combination of adjectives is said to measure the emotional state, these millemetre scores are transformed by the calculation of a weighted sum into one single score for the QBA. This is done according to the formula

$${\text{QBA score }} = \, - 4.5367 \, + \sum\limits_{k - 1}^{20} {{\text{w}}_{k} \cdot{\text{N}}_{k} }$$

Thereby, w_k_ represents the attributed weight to each of the 20 adjectives (the given term k) and N_k_ is the value in millimetres that was obtained on the farm for each of the 20 adjectives (the given term k). This procedure was done following strictly the information provided in the Welfare Quality protocol for growing pigs, in which the attributed weights are also listed (Welfare Quality^®^^2009^). Given this formula, the attributed weights and the scale ranging from 0–125 mm, the QBA score can take theoretical values ranging from −15.60 to 8.61.

#### Behaviour observations (BO)

In the stable, after the QBA, BO in the form of instantaneous scan sampling were performed on three other viewpoints. Depending on the size of the pens, it was possible to observe two to four pens at each viewpoint (40–60 animals). First, the pigs in the pens under surveillance were chased up and then they had 5 min time for calming down. During this time, coughing and sneezing was counted. Afterwards, the animals were scanned for a total time of 10 min at each viewpoint. A scan was made every 2 min and the pigs were then sorted into the categories positive social behaviour, negative social behaviour, pen investigation, use of enrichment material, other active behaviour or resting.

The results of the BO were expressed as performed behaviour in percent of the total active behaviour. Thereby, positive and negative social behaviour were expressed together as total social behaviour and negative social behaviour was also presented individually.

#### Human Animal Relationship Test (HAR)

In the following protocol assessment, ten randomly chosen pens were entered and initially, the reaction of the animals towards the intruder was evaluated by a Human Animal Relationship Test. As the animals in the pens used for the assessment of a panic reaction towards an intruder were also the pigs assessed for all IP measurements, it was decided to enter the pens one after the other to minimize mutual interference. This might have influenced the reaction of the pigs towards the second person, though. Therefore, with the present study design, it was not possible to evaluate the interobserver reliability of the HAR.

#### Individual parameters (IP)

After the HAR, the pigs in the pens entered were scored for a variety of IP, e.g. wounds, manure on the body, tail lesions and bursitis, whereby only one side of the pigs was considered. The IP were either scored using a three point scale (0 = absent, 1 = light affection, 2 = strong affection) or else a two point scale (0 = absent, 2 = present). The complete list of parameters, their definitions and the slotting criteria are presented in Table 1. Going in accordance with the protocol, some resource based parameters were also taken into account, e.g. the number, functioning and cleanliness of the drinkers as parameter for the absence of prolonged thirst. Further, the sizes of the pens were measured and the weight of the animals was estimated to determine the space per 100 kg. The mortality rate and the percentages of animals affected by pneumonia, pleurisy, ascites and pericarditis registered by the slaughterhouse were asked from the farmer as well as whether and how management procedures such as tail docking and castrating are carried out.

**Table 1:** Quantitative animal based measures with scoring scale and definition (Welfare Quality^®^, 2009)

Animal based measure	Score	Definition
Body condition	0	Good body condition
	2	Thin: visible spine, hip, pin bones
Bursitis	0	No evidence of bursae/swelling
	1	One/several small bursae on the same leg or one large bursa
	2	Several large bursae on the same leg or one extremely large or eroded bursa
Manure	0	<20 % of body surface soiled with faeces
	1	20–50 % of body surface soiled with faeces
	2	>50 % of body surface soiled with faeces
Huddling	0	Pig lying with <50 % of its body on top of other pig
	2	Pig lying with >50 % of its body on top of other pig
Shivering	0	No vibration of any body part
	2	Vibration of any body part
Panting	0	Normal breathing
	2	Rapid breath in short gasp
Wounds	0	<4 lesions on all zones of the body
	1	4–10 lesions on one or more zones of the body
	2	≥10 lesions on two zones of body or one zone > 15 lesions
Tail biting	0	No evidence of tail biting
	2	Evidence of tail biting
Lameness	0	Normal gait or slight lameness or abnormality in gait
	1	Severely lame, weight bearing on affected limb
	2	No weight bearing on one limb or unable to walk
Pumping	0	No evidence of laboured breathing
	2	Laboured breathing
Scouring	0	No liquid manure visible in pen
	1	Some liquid manure in some areas of pen
	2	All faeces visible inside pen are liquid
Skin Condition	0	All skin of normal colour and texture
	1	0–10 % of skin has an abnormal colour or texture
	2	>10 % of skin has abnormal colour or texture
Hernia	0	No hernia/rupture
	1	Small hernia/rupture
	2	Hernia/rupture touching the floor or with bleeding lesion
Twisted snout	0	No evidence of twisted snout
	2	Evidence of twisted snout
Rectal prolapse	0	No evidence of rectal prolapse
	2	Evidence of rectal prolapse
Coughing	n	Number of coughs
Sneezing	n	Number of sneezes
Human Animal Relation	0	≤60 % showing a panic response
	2	>60 % of the pigs fleeing, facing away or huddled in corner of pen
Negative social behaviour	%	Aggressive behaviour or any behaviour with a response from the disturbed animal or any tail in mouth behaviour
Positive social behaviour	%	Sniffing, nosing, licking and moving gently away from the animal without an aggressive or flight reaction from this individual
Pen investigation	%	Sniffing, nosing, licking all features of pen
Use of enrichment material	%	Exploration towards straw and other suitable enrichment material

The IPs were analysed as the percentage of animals sorted into the corresponding category (e.g. bursitis category 0: 50 %, bursitis category 1: 40 %, bursitis category 2: 10 %). Thereby, the categories were treated as independent variables and were compared individually. For instance, bursitis 0, bursitis 1 and bursitis 2 were analysed separately although they are dependent on each other such that if one animal was not scored into category 0, it had to be scored into one of the other two categories.

### Statistics and reliability and agreement parameters

Results were compared at parameter level without further aggregation into criteria or principle scores. Furthermore, all results were expressed at farm level, which is reasonable, since the samples of animals were taken randomly to give an overview of the assessed farm (Welfare Quality^®^^2009^). The values of the recorded parameters in percent respectively in millimetres achieved from each of the observers were then compared and evaluated for their reliability.

As the comparison between observer A and B respectively observer A and C led basically to the same results, the observations were aggregated as one table of observations. Therefore, the results are displayed as comparison between observer A and observer BC, whereby the column observer BC includes the results of observer B and observer C.

 For statistical analysis, different reliability and agreement parameters were calculated using the statistic program SAS 9.2 (S.A.S. Institute 2008) or R (Version 2.11.1) (Venables and Smith 2010). In the case of the Spearman Rank Correlation Coefficient, the Procedure Proc Corr in SAS 9.2 (S.A.S. Institute 2008) was used. For the other parameters (Intraclass Correlation Coefficient (ICC), Smallest Detectable Change (SDC), Limits of Agreement (LoA), the IRR package (Gamer et al. 2012) for R (Version 2.11.1) was used for calculation.

#### Spearman Rank Correlation Coefficient (RS)

The RS, which is a non-parametric technique for the evaluation of the degree of linear correlation between two variables, is often used in animal welfare science (Dalmau et al. 2010). However, it does not directly compare the values obtained, but solely the rank order (Dohoo et al. 2003). The values can range from −1 to 1, whereat correlation is better the closer the value is to 1. Negative values indicate negative correlations. According to Martin and Bateson (2007), RS equal to or greater than 0.4 is interpreted as acceptable correlation and equal to or greater than 0.7 as good correlation.

#### Intraclass Correlation Coefficient (ICC)

The ICC is based on an analysis of variance and assesses reliability by putting into proportion the variance of the same subject (farm visits, observers) to the total variance of all measures and subjects (de Vet et al. 2006). It is a common and useful parameter for the assessment of reliability in medical and psychological studies (Weir 2005) and a more frequent use in other subjects such as animal welfare studies is strongly encouraged (McGraw and Wong 1996).

For the fundamental analysis of variance the following two way model was assigned according to Shrout and Fleiss (1979):

$${{\rm{X}}_{{\rm{ijK}}}} = \mu + {\alpha _{\rm{i}}} + {\beta _{\rm{j}}} + {(\alpha \cdot \beta )_{{\rm{ij}}}} + {\varepsilon _{{\rm{ijk}}}}$$

with x_ijk_ being the measured value, µ the general average value, α_i_ the fixed effect of the difference between the measurement objects (farms), β_j_ the random effect of the observers, (α * β)_ij_ the interaction effect between observers and objects and ε_ijk_ as the general error term. ICC was calculated according to the formula of agreement (Shrout and Fleiss 1979):

$${\text{ICC}} = \frac{{\sigma ({\text{Objects}})}}{{\sigma^{2} ({\text{Objects}}) + \sigma^{2} ({\text{Observers}}) + \sigma^{2} ({\text{residual}})}}$$

with σ^2^ describing the variance of the study objects, the observers and the residual variance, respectively.

According to this formula, ICC can take values between 0 and 1, thereby, a value of 0 describes a total lack of reliability and a value of 1 describes perfect reliability (de Vet et al. 2006). As proposed by McGraw and Wong (1996), an ICC equal to or greater than 0.4 was interpreted as acceptable reliability and an ICC greater than or equal to 0.7 as good reliability.

#### Smallest Detectable Change (SDC)

The SDC is an expression of the measurement error. The measurement error contains in this case the variance of the observers and the residual variance and is achieved from the above named formulas. SDC is calculated according to de Vet et al. (2006) by the formula

$${\text{SDC }} = \, 1.96 \cdot \surd 2 \cdot \left( {\sigma^{2}_{{ ( {\text{observers)}}}} + \sigma^{2}_{{ ( {\text{residual)}}}} } \right).$$

It gives the smallest change in the score that can be detected with the instrument despite the measurement error. The measurement unit of the SDC is in accordance with the measurement unit of the parameters under surveillance, thus, in the present case it is expressed in percent. Based on the interpretation of the simple agreement coefficient in de Vet et al. (2006), a SDC lesser than or equal to 0.1 was interpreted as acceptable agreement. For unification purposes of the presentation format, the differences of the QBA scores are also expressed in differences in percent.

#### Limits of Agreement (LoA)

LoA was also calculated according to de Vet et al. (2006) by the formula

$${\text{LoA }} = {\text{ mean }} \pm \, 1.96 \cdot \left( {\surd 2 \cdot \sigma^{2}_{{ ( {\text{residual)}}}} } \right).$$

In this case, $\alpha_{\text (residual)}^{2}$ contains also the variance of the observers $(\alpha_{\text (observers)}^{2})$. The LoA, which was first introduced by Bland and Altmann (1986) calculates the range of the difference between two sets of measurement values and is in this study expressed as the relative frequency between −1 and 1. The direction of −1 would be differences according to higher values obtained by observer BC and the direction of 1 due to higher values achieved by observer A. Again, interpretation was based on the simple agreement coefficient of de Vet et al. (2006) and thus, an interval lesser than or equal to −0.1 to 0.1 was interpreted as acceptable agreement. The plot of the LoA, namely the plot of difference between the means of two measurements against the average prevalence helps to determine the range of errors (Temple et al. 2013).

## Results

### Protocol assessments

#### Qualitative Behaviour Assessment (QBA)

The mean values obtained by each observer for each of the adjectives as well as the mean of the weighted sum and the corresponding agreement parameters are shown in Table 2. No agreement was found in the direct comparison of millimetre scores in any of the adjectives. Even if good agreement was achieved for the RS and the ICC, concerning for instance the term ‘relaxed’, the values of the SDC and LoA indicated low agreement. However, the overall QBA scores obtained by the calculation of a weighted sum had acceptable values in the calculation of RS and ICC and exceeded the predefined limits for acceptability for the SDC and LoA only narrowly by one percent point.

**Table 2:** Mean values [mm] of the two observers for the adjectives of the Qualitative Behaviour Assessment (QBA) as well as the mean of the weighted sum and corresponding reliability parameters

Adjective	Observer A	Observer BC	RS	ICC	SDC	LoA
Active	87.1	65.9	0.50	0.30	0.38	−0.05 to 0.45
Relaxed	49.2	43.2	0.61	0.64	0.32	−0.28 to 0.41
Fearful	14.6	10.4	0.06	0.00	0.26	−0.20 to 0.30
Agitated	41.2	28.2	0.64	0.40	0.39	−0.25 to 0.49
Calm	42.3	44.9	0.15	0.70	0.28	−0.40 to 0.23
Content	55.0	49.9	0.35	0.36	0.25	−0.21 to 0.30
Tense	33.5	18.2	0.45	0.23	0.37	−0.12 to 0.48
Enjoying	33.3	76.7	0.15	0.21	0.27	−0.40 to 0.22
Frustrated	18.9	12.0	0.43	0.22	0.25	−0.10 to 0.35
Bored	17.1	17.8	0.15	0.36	0.28	−0.32 to 0.30
Playful	24.4	29.8	0.28	0.36	0.32	−0.40 to 0.33
Positively occupied	61.6	96.3	0.43	0.26	0.34	−0.15 to 0.45
Listless	16.3	9.3	0.19	0.26	0.26	−0.21 to 0.31
Lively	54.5	62.2	0.56	0.00	0.49	−0.48 to 0.40
Indifferent	18.1	28.9	0.40	0.38	0.31	−0.35 to 0.38
Irritable	25.1	13.8	0.35	0.26	0.31	−0.12 to 0.42
Aimless	25.6	18.5	0.04	0.38	0.34	−0.25 to 0.43
Happy	46.5	50.1	0.04	0.19	0.25	−0.32 to 0.20
Distressed	7.8	9.7	0.10	0.24	0.21	−0.23 to 0.17
Sociable	66.4	58.9	0.30	0.20	0.31	−0.28 to 0.39
Weighted sum	−0.80	−0.48	0.62	0.62	0.11	−0.11 to 0.09

*RS* Spearman Rank Correlation Coefficient, *ICC* Intraclass Correlation Coefficient, *SDC* Smallest Detectable Change, *LoA* Limits of Agreement

#### Behaviour observations (BO)

On average, the observers sorted similar percentages of animals into the dedicated behavioural categories. This agreement could also be obtained in the calculation of the agreement parameters, which achieved for all behavioural categories acceptable to good values. Mean values and statistical parameters are presented in Table 3.

**Table 3:** Mean prevalences of the categories of the behavioural observations as well as individual parameters with a prevalence greater than 0.5 % assigned by observer A and BC and corresponding reliability parameters

Parameter	Cate-gory	Mean prevalence observer A	Mean prevalence observer BC	RS	ICC	SDC	LoA
Social behaviour		9.8	10.5	0.45	0.50	0.09	−0.09 to 0.03
Negative social behaviour		3.1	2.0	0.58	0.40	0.06	−0.05 to 0.03
Use of enrichment material		5.2	4.3	0.75	0.68	0.06	−0.03 to 0.03
Pen investigation		25.1	29.8	0.40	0.48	0.10	−0.10 to 0.10
Bursitis	0	55.3	55.1	0.26	0.0	0.43	−0.40 to 0.49
	1	44.2	38.0	0.10	0.0	0.35	−0.37 to 0.40
	2	0.7	6.1	0.01	0.0	0.19	−0.22 to 0.21
Manure	0	77.5	77.6	0.69	0.78	0.10	−0.10 to 0.10
	1	14.2	17.1	0.44	0.71	0.09	−0.10 to 0.09
	2	8.3	4.5	0.45	0.59	0.09	−0.09 to 0.08
Wounds	0	86.7	87.8	0.58	0.41	0.10	−0.10 to 0.10
	1	8.7	10.3	0.46	0.59	0.10	−0.09 to 0.10
	2	1.2	1.3	0.40	0.84	0.03	−0.02 to 0.01
Tail biting	0	96.2	97.3	0.43	0.88	0.05	−0.01 to 0.02
	2	3.8	2.7	0.43	0.88	0.05	−0.01 to 0.02
Lameness	0	99.3	98.4	0.64	0.58	0.05	−0.07 to 0.05
	1	0.5	0.6	0.46	0.40	0.02	−0.01 to 0.0
Skin condition	0	96.3	97.6	0.66	0.44	0.09	−0.05 to 0.07
	1	1.1	1.7	0.44	0.58	0.08	−0.06 to 0.05
Hernia	0	99.3	98.6	0.61	0.70	0.05	−0.01 to 0.02
	1	0.6	0.7	0.50	0.60	0.01	−0.01 to 0.01
Coughs/pig	n	9.6	9.1	0.70	0.98	0.08	−0.03 to 0.06
Sneezes/pig	n	2.9	3.4	0.64	0.71	0.04	−0.03 to 0.01

*RS* Spearman Rank Correlation Coefficient, *ICC* Intraclass Correlation Coefficient, *SDC* Smallest Detectable Change, *LoA* Limits of Agreement

#### Individual parameters (IP)

The parameters panting, shivering, pumping, twisted snout, rectal prolapse, poor body condition and hernia category 2 did not occur at all and the parameters huddling, scouring and lameness category 2 were observed only to a prevalence of less than 0.05 %, which would make an assumption about their reliability untrustworthy.

The remaining parameters, however, were recorded with a prevalence of greater than 0.5 % and thus reliability could be assessed in a sensible way. The mean prevalences of the parameters assigned by observer A and BC, respectively, and the corresponding agreement and reliability parameters for these measures can be found in Table 3. Most of the parameters proved acceptable to good agreement. However, the reliability parameters for bursitis of all categories indicated non-satisfactory agreement.

## Discussion

### Reliability and agreement parameters

Different agreement and reliability parameters were chosen for the calculation and analysis of the reliability, as each parameter has its own weaknesses and benefits and as the interpretation of only one parameter can easily lead to misinterpretations.

Correlation Coefficients such as the RS and the ICC are measures of reliability as they evaluate the degree to which study objects can be distinguished from each other despite the measurement error. The main limitation of these parameters, however, is that they are strongly dependent on the total variance of the assessed objects. These reliability parameters achieve higher scores if the variability is large, meaning that there are great differences among the study objects and they can become very small despite good reliability if the study objects are very similar to each other (de Vet et al. 2006). This dependency on the total variance has to be taken into account when analysing reliability parameters to avoid misinterpretations (Wirtz and Caspar 2002). Although the SDC and the LoA are mathematically derived from the ICC, they are parameters of agreement, since they assess (by estimating the measurement error) how close results of repeated measures are. Therefore, they are not influenced by the variance of the assessed population. The problem with these agreement parameters is, however, that interpretation of the outcomes is highly subjective. Due to these individual problems of each parameter, it was deemed necessary to calculate and interpret a combination of reliability and agreement parameters, as advised by de Vet et al. (2006).

### Protocol assessments

The prevalences obtained for the assessments carried out in terms of the protocol were mostly in accordance to those found in previous studies (Temple et al. 2011b, 2012, 2013).

#### Qualitative Behaviour Assessment (QBA)

In the QBA, no congruent agreement between the assessors could be found when comparing the millimetre length assigned to each adjective at farm level. This stands in contrast to the findings of Wemelsfelder and Millard (2009), who used solely Kendall’s Tau as reliability parameter. In the present study, for some adjectives, the RS and the ICC indicated a good agreement in none of the cases did all four parameters (RS, ICC, SDC, LoA) suggest this conclusion. The calculation of an overall QBA score with a weighted sum presented better agreement than the comparison of the single adjectives, although still not acceptable concerning the SDC and LoA. Nevertheless, this suggests that the QBA might be a reliable method after reconsideration of the weighted sum, which is, up to date, not totally transparent or else adjustment in the form that those adjectives with specifically low reliability are replaced by better ones.

#### Behaviour observations (BO)

The BO revealed a good to moderate agreement and uniformity across all calculated reliability parameters. Thus, this method has a good interobserver reliability. This indicates that trained observers sort the animals into the same categories.

#### Individual parameters (IP)

The classes of those IP with three categories were assessed independently of each other, ignoring the interaction between the categories, which was also done by Temple et al. (2011a). This approach was chosen according to the hypothesis that categorisation between 0 and 2 might be of good agreement while the definition of 1 might have caused problems.

The interobserver reliability of those IP that appeared with a prevalence of greater than 0.5 % was in general acceptable to good. Manure and wounds of category 1 were only just acceptable, while category 2 values especially for wounds were of clearly better reliability. Although this proves that the classification into just two categories would be more robust, an exclusion of category 1 is not recommended since three categories provide a higher informative value, with light affections also being taken into account. Furthermore, reliability was still acceptable.

The observers did not agree in the assessment of the parameter bursitis. Temple et al. (2013) also stated an insufficient reliability for this parameter, in contrast Forkman and Keeling (2009) found a good reliability. However, they used the five scale scoring system for bursitis of Lyons et al. (1995) and therefore, it is not directly comparable to our study, as in terms of the Welfare Quality^®^ protocol, a three point scale was used (Veissier et al. 2013). The low reliability can also be explained by the fact that in terms of the ‘Welfare Quality^®^ Animal Welfare Assessment’ protocol, this parameter is assessed visually as a swelling in the region of the joints of the legs. When the animals are moving fast, the legs are dirty or the stable is relatively dark, our practical experience shows that this can be quite hard to assess. This corresponds to the findings of Veissier et al. (2013) who stated that the number of animals per pen, the stocking density, the dirtiness of pigs and the light intensity in the stable influences the recording of measures. To ensure the categorisation by palpating, as proposed during training sessions, was often not possible on such a great number of pigs that cannot be fixated. Furthermore, other causes of swellings in the region of the joint, e.g. haematoma or bacterial infection leading to increased synovial fluids in the joints (Plonait et al. 2004) cannot be differentiated securely by visual assessment. Bursitis category 2 was of slightly better reliability thus indicating a clearer definition. However, the present results indicate that the parameter bursitis as it is presently defined in the Welfare Quality^®^ protocol for growing pigs is not useful for assessing the comfort around resting as it cannot be assessed in a reliable manner. Therefore, our suggestion is that for the reliable assessment of comfort around resting, other parameters should be taken into account or else a revised definition of bursitis in a manner that can be assessed reliably.

## Conclusion

The aim of the present study was to assess the interobserver reliability of the ‘Welfare Quality^®^ Animal Welfare Assessment Protocol for Growing Pigs’. No sufficient reliability was found in terms of the QBA. However, the calculation of a weighted sum suggests that it might be a suitable method after adjustment. BO in the form of instantaneous scan sampling as a parameter for the assessment of social and other behaviour turned out to provide for a good reliability. In general, good reliability was assigned to the IP. The only exception has to be made for bursitis as the parameter for comfort around resting for which a better definition and assessment method or another suitable parameter are probably needed. Some parameters occurred only rarely or not at all, thus making an assumption about their reliability meaningless. In general, the ‘Welfare Quality^®^ Animal Welfare Assessment Protocol for Growing Pigs’ could be a promising approach for a feasible and reliable welfare assessment tool after revision of some parameters.

## Abbreviations

BO: direct behaviour observations
HAR: Human Animal Relationship Test
ICC: Intraclass Correlation Coefficient
IP: individual parameters
LoA: Limits of Agreement
QBA: Qualitative Behaviour Assessment
RS: Spearman Rank Correlation Coefficient
SDC: Smallest Detectable Change
